# From Dysbiosis to Hepatic Inflammation: A Narrative Review on the Diet-Microbiota-Liver Axis in Steatotic Liver Disease

**DOI:** 10.3390/microorganisms13020241

**Published:** 2025-01-23

**Authors:** Andrea Pasta, Elena Formisano, Francesco Calabrese, Elisa Marabotto, Manuele Furnari, Giorgia Bodini, Maria Corina Plaz Torres, Livia Pisciotta, Edoardo Giovanni Giannini, Patrizia Zentilin

**Affiliations:** 1 Gastroenterology Unit, Department of Internal Medicine, University of Genoa, 16132 Genoa, Italy; andreapasta93@gmail.com (A.P.); calabrese.francesco.93@gmail.com (F.C.); elisa.marabotto@unige.it (E.M.); manuele.furnari@unige.it (M.F.); giorgia.bodini@unige.it (G.B.); mariacorina.plaztorres@edu.unige.it (M.C.P.T.); egiannini@unige.it (E.G.G.); 2 Dietetics and Clinical Nutrition Unit, Department of Internal Medicine, University of Genoa, 16132 Genoa, Italy; elena.formisano@hsanmartino.it (E.F.); livia.pisciotta@unige.it (L.P.); 3 IRCCS Ospedale Policlinico San Martino, 16132 Genoa, Italy

**Keywords:** gut microbiota, gut-liver axis, dysbiosis, steatotic liver disease

## Abstract

The gut microbiota has emerged as a critical player in metabolic and liver health, with its influence extending to the pathogenesis and progression of steatotic liver diseases. This review delves into the gut-liver axis, a dynamic communication network linking the gut microbiome and liver through metabolic, immunological, and inflammatory pathways. Dysbiosis, characterized by altered microbial composition, contributes significantly to the development of hepatic steatosis, inflammation, and fibrosis via mechanisms such as gut barrier dysfunction, microbial metabolite production, and systemic inflammation. Dietary patterns, including the Mediterranean diet, are highlighted for their role in modulating the gut microbiota, improving gut-liver axis integrity, and attenuating liver injury. Additionally, emerging microbiota-based interventions, such as fecal microbiota transplantation and bacteriophage therapy, show promise as therapeutic strategies for steatotic liver disease. However, challenges such as population heterogeneity, methodological variability, and knowledge gaps hinder the translational application of current findings. Addressing these barriers through standardized approaches and integrative research will pave the way for microbiota-targeted therapies to mitigate the global burden of steatotic liver disease.

## 1. Introduction

Steatotic liver disease represents a spectrum of conditions characterized by the accumulation of fat in the liver, with varying degrees of metabolic and inflammatory involvement [1,2,3]. Historically termed non-alcoholic fatty liver disease (NAFLD), this condition encompasses a wide range of presentations, from simple hepatic steatosis to more advanced states such as non-alcoholic steatohepatitis (NASH) [4]. Recent advancements in understanding its pathophysiology have prompted a paradigm shift in classification. The term metabolic-associated fatty liver disease (MAFLD) was introduced to emphasize its strong association with metabolic dysfunction, providing a diagnostic framework that aligns with the systemic and multifactorial nature of the disease [5]. In parallel, the transition to metabolic-associated steatotic liver disease (MASLD) within clinical and research contexts reflects evolving efforts to refine terminology and incorporate emerging insights, including genetic and environmental contributions [1,6,7,8]. These refinements underscore the complexity of steatotic liver disease and its significant public health burden [9].

The global prevalence of steatotic liver disease is alarmingly high, affecting over 30% of the adult population, with higher rates in individuals with obesity, type 2 diabetes, and metabolic syndrome [10]. These staggering figures highlight its significance as a leading cause of liver-related morbidity and mortality [10,11]. Moreover, the interplay between systemic metabolic dysfunction and liver health places steatotic liver disease at the crossroads of various chronic diseases, necessitating multidisciplinary management strategies [12].

An emerging and critical area of research involves the role of the gut microbiota in metabolic and liver diseases [13]. The gut-liver axis, a bidirectional communication network linking the gut microbiome and liver through metabolic, immunological, and inflammatory pathways, has garnered significant attention [14,15]. Dysbiosis, or the imbalance in gut microbial composition, has been implicated in the pathogenesis and progression of steatotic liver disease [16]. Altered gut permeability, microbial metabolites, and systemic inflammation are among the mechanisms through which the microbiota exerts its influence on liver health [17].

This review aims to explore the intricate relationship between the gut microbiota and MASLD. By synthesizing current evidence, it seeks to explain the mechanisms underlying this interaction and its implications for disease progression, therapeutic interventions, and overall metabolic health. Understanding the microbiota–MASLD connection holds promise for novel diagnostic and therapeutic approaches that may transform the management of steatotic liver diseases.

## 2. Methodology

A thorough literature search was performed to identify studies exploring the interplay between gut microbiota and steatotic liver diseases, including MASLD. Key topics included the gut-liver axis, microbial dysbiosis, microbial metabolites, dietary patterns, and their impact on liver health. The search focused on studies addressing both the mechanistic and clinical aspects of these relationships, aiming to synthesize a comprehensive understanding of current knowledge. Studies published in English over the past 15 years were considered, with exceptions made for earlier landmark studies crucial to the historical understanding of the topic.

Relevant literature was selected based on its contribution to understanding the composition and function of the gut microbiota, its role in the progression or management of steatotic liver disease, and the impact of diet on the gut-liver axis. Clinical, preclinical, and mechanistic studies were included, while studies not published in English or focusing on unrelated liver or gastrointestinal disorders were excluded. The selected evidence was critically analyzed and synthesized into a comprehensive narrative format highlighting the interactions between the gut microbiota, dietary factors, and liver health.

## 3. The Gut-Liver Axis

The gut-liver axis represents a critical bidirectional communication system connecting the gut, its microbiota, and the liver [18]. This relationship is primarily facilitated by the portal vein, which delivers gut-derived products directly to the liver, and by the liver’s feedback to the gut via bile acids and immunological mediators [19]. Under normal conditions, the intestinal barrier, composed of mucus, epithelial cells, and immune defenses, plays a pivotal role in maintaining this equilibrium [20].

The mucus layer acts as the first line of defense, separating gut microbiota from direct contact with epithelial cells. It consists of an outer layer, colonized by microbes, and an inner sterile layer, enriched with antimicrobial peptides and microbiota-excluding proteins. These structural components not only prevent microbial invasion but also foster a controlled environment for nutrient absorption and immune surveillance [21].

Bile acids, synthesized in the liver and released into the gut, further regulate gut-liver communication. Beyond their role in lipid digestion, bile acids interact with the microbiota and intestinal epithelium via nuclear receptors such as the farnesoid X receptor (FXR) [22]. This signaling modulates epithelial barrier integrity and systemic metabolic functions, ensuring balanced microbial composition and preventing excessive microbial translocation [23].

Additionally, the gut-associated lymphoid tissue (GALT) enhances immune-mediated control, producing immunoglobulin A to neutralize potential pathogens [24]. This immunological layer works in synergy with epithelial tight junctions to prevent the systemic dissemination of harmful microbial products while permitting nutrient transfer [25].

In homeostasis, these mechanisms ensure a symbiotic relationship where microbial metabolites such as short-chain fatty acids (SCFAs) support colonic health and systemic metabolic regulation. This intricate balance highlights the gut-liver axis as a cornerstone of systemic health, emphasizing the importance of its integrity in preventing liver and systemic diseases [26].

## 4. Gut Microbiota Signature in Health and Steatotic Liver Disease

The concept of a “microbiota signature” refers to the distinct composition and functional profile of the gut microbial community, which can reflect the health status of an individual and provide insights into disease mechanisms [27,28]. These signatures are shaped by interactions between host genetics, diet, environment, and microbial ecosystems [26,27]. In a healthy state, the gut microbiota signature is marked by diversity, resilience, and the presence of key functional taxa that support metabolic and immune balance [29]. Conversely, deviations in this signature—characterized by reduced diversity, loss of beneficial microbes, and overgrowth of pathobionts—are strongly associated with various disease states [30,31], including metabolic and steatotic liver disorders [32].

The gut microbiota plays a central role in host physiology by influencing nutrient absorption, immune regulation, and systemic metabolic pathways [33]. Through the gut-liver axis, the microbiota directly impacts liver health by modulating bile acid metabolism, intestinal permeability, and inflammatory responses. Dysbiosis, or an imbalance in the microbial community, disrupts this equilibrium and contributes to the pathogenesis of conditions such as MASLD and MASH [34].

In healthy subjects, the gut microbiota exhibits high diversity and stability, dominated by the phyla *Firmicutes* and *Bacteroidetes* [33]. Key bacterial genera, including *Faecalibacterium* and *Ruminococcus*, are associated with the production of SCFAs, such as butyrate, acetate, and propionate, which enhance intestinal barrier function and regulate host energy metabolism [35]. Other beneficial genera, such as *Alistipes* and *Akkermansia*, contribute to anti-inflammatory signaling and gut epithelial integrity [36,37]. The gut microbiota also influences bile acid metabolism, transforming primary bile acids into secondary forms via microbial enzymes. This process regulates lipid absorption, cholesterol homeostasis, and microbial composition. Interaction with nuclear receptors further underscores the microbiota’s systemic impact [37,38]. Recent studies have highlighted significant differences in the gut microbiota composition between MAFLD patients and healthy individuals [37,39,40]. A consistent finding is the reduction in microbial diversity among MAFLD patients, reflecting a disrupted gut ecosystem. While alpha diversity metrics, such as Shannon and Simpson indices, do not always exhibit stark differences, beta diversity analyses have revealed distinct microbial compositions between healthy and diseased groups [37,41].

In this context, specific bacterial taxa are frequently associated with MAFLD. Notably, the abundance of *Ruminococcus obeum* and *Alistipes* is significantly reduced in MAFLD patients compared to healthy controls [37,42,43]. *Alistipes*, a genus known for its anti-inflammatory properties, has been inversely correlated with markers of liver injury, including serum glucose, gamma-glutamyl transferase, and alanine aminotransferase [44,45]. This suggests a protective role for these bacteria in metabolic health [46]. Conversely, genera such as *Dorea*, *Lactobacillus*, and *Megasphaera* are often enriched in individuals with MAFLD [37]. *Dorea* has been implicated in pro-inflammatory processes and is frequently associated with conditions such as obesity and NASH [47,48]. Similarly, *Lactobacillus* species, despite their common use as probiotics, may exhibit strain-dependent effects, with certain strains promoting inflammatory pathways [49]. *Megasphaera*, known for its ability to ferment carbohydrates into SCFAs, is enriched in obese populations, potentially contributing to energy harvest and adiposity [50,51]. Bacterial genera such as *Bacteroides vulgatus* and *Ruminococcus gnavus* are also implicated in MAFLD. *Bacteroides vulgatus* exhibits lower relative abundance in MAFLD patients [52], while *Ruminococcus gnavus* is positively associated with increased waist circumference and elevated triglyceride levels, highlighting its potential role in lipid metabolism dysregulation [53]. Another critical aspect is the correlation between gut microbiota alterations and liver enzyme abnormalities in MAFLD subgroups [37]. Patients with elevated liver enzymes often show a further shift in microbial composition, with a notable decrease in beneficial taxa [54].

An important study by Frost et al. [36], conducted as part of the longitudinal Study of Health in Pomerania (SHIP), examined changes in gut microbiota composition and diversity over a five-year period between SHIP-2 and SHIP-3. This analysis focused on the relationship between microbiota stability and metabolic disorders, offering insights into early microbial changes as predictors of disease onset. Overall, the gut microbiota remained largely stable across the population during the study period. Dominant taxa, such as *Bacteroides*, *Prevotella*, and *Faecalibacterium*, persisted over time, reflecting the resilience of the gut ecosystem. However, this stability was not uniform, as specific changes were observed in individuals affected by metabolic disorders. A slight reduction in microbial diversity, particularly in species richness, was noted, accompanied by shifts in community structure linked to conditions such as metabolic liver diseases and diabetes mellitus. Participants with fatty liver disease or diabetes mellitus displayed a significantly increased presence of facultative pathogens, including *Enterobacteriaceae*, *Escherichia/Shigella*, and *Citrobacter*. One of the most intriguing findings of the study was the ability of early microbial changes to predict disease development. Individuals who were diagnosed with fatty liver disease or diabetes mellitus at SHIP-3 already exhibited distinct gut microbiota alterations at SHIP-2, even before the clinical onset of the diseases. Specific taxa, such as *Clostridium XIVa*, were found to be associated with pathways involved in lipid biosynthesis, suggesting a potential role in promoting hepatic lipid accumulation [36]. These early changes highlight the potential of the gut microbiota as a predictive biomarker for metabolic diseases, offering opportunities for early intervention.

**Figure 1:**
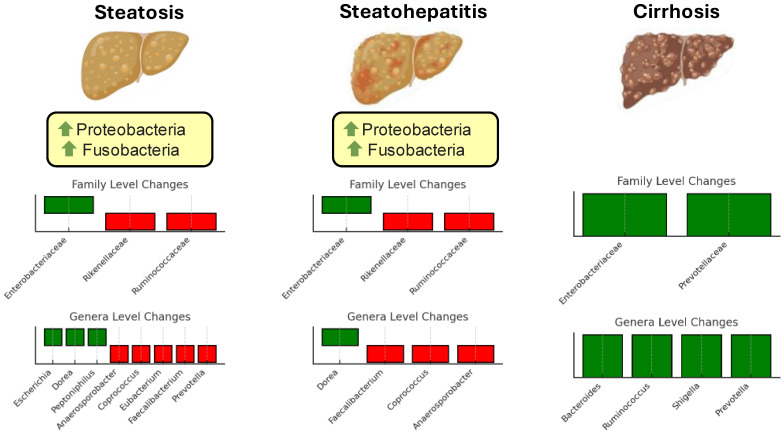
Changes in gut microbiota composition at different stages of steatotic disease progression. The figure illustrates the alterations in microbial taxa across three stages: simple steatosis, steatohepatitis, and cirrhosis. Family-level changes are shown in the top bar plots for each stage, while genus-level changes are displayed in the lower bar plots. Green bars represent taxa with increased abundance, and red bars indicate taxa with decreased abundance. Green arrows signify an increase in abundance at the phylum level.

While substantial progress has been made in identifying microbial patterns associated with metabolic and steatotic liver diseases, several challenges remain. The data are often limited and derived from small, heterogeneous cohorts, making generalizability difficult. Moreover, findings from different studies sometimes appear contradictory, likely reflecting variations in study design, population characteristics, and methodologies used for microbiota analysis. For instance, while some taxa are consistently associated with protective or pathogenic roles, others exhibit context-dependent effects, influenced by host factors such as diet, genetics, and disease stage. Additionally, the complex and dynamic nature of the gut microbiota complicates efforts to establish definitive causal links between specific microbial changes and disease progression [55]. Figure 1 illustrates changes in the gut microbiota composition at different stages of liver disease progression.

## 5. Mechanistic Links Between Microbiota and MASLD

The interplay between intestinal microbial communities and liver physiology is primarily mediated through microbial metabolites, immune signaling mechanisms, and inflammatory pathways [56].

In an old but significant animal experiment, researchers demonstrated that the gut microbiota plays a crucial role in regulating fat storage and energy balance. By colonizing germ-free mice with microbiota from conventionally raised mice, they observed a 60% increase in body fat content and insulin resistance within 14 days, despite reduced food intake [57]. This study identified the microbiota’s power to promote monosaccharide absorption and hepatic triglyceride production while suppressing the intestinal expression of fasting-induced adipocyte factor (Fiaf), a lipoprotein lipase inhibitor [57,58]. In this context, in obese individuals, the microbiota is characterized by an increased abundance of Firmicutes, which efficiently ferment dietary polysaccharides into SCFAs, such as butyrate [59,60]. This fermentation increases caloric yield and supports lipid synthesis and storage in adipocytes [59]. Notably, butyrate, a key product of Firmicutes, has anti-inflammatory properties, including the maintenance of gut barrier integrity and the modulation of immune responses [61]. These properties may counteract inflammatory processes commonly associated with obesity. Metagenomic analyses reveal that the Firmicutes-enriched microbiota in obesity is associated with elevated levels of enzymes involved in carbohydrate fermentation and SCFA production, as well as reduced energy loss in feces [62]. This functional profile highlights the microbiota’s dual role: enhancing energy harvest while potentially mitigating inflammation through butyrate production, reflecting a complex balance in host metabolism [37,63].

Another mechanism contributing to steatotic liver disease involves the production of ethanol by gut microbiota. In a recent study, high-alcohol-producing strains of Klebsiella pneumoniae (HiAlc Kpn) were identified as a significant contributor to NAFLD pathogenesis. These strains generated ethanol within the gut, which, when transferred to mice, induced NAFLD, characterized by hepatic steatosis and inflammation. The study highlights the potential of microbiota-targeted interventions to reduce endogenous alcohol production and its harmful effects on the liver [64]. Moreover, it was found that microbial ethanol production could be detected in the portal vein, with concentrations significantly higher in individuals with NAFL and NASH compared to those without steatosis. Intervention with antibiotics drastically reduced ethanol levels, confirming the microbial origin [64]. This evidence underscores the role of microbial ethanol as a dietary and metabolic factor influencing liver health and the progression of NAFLD.

A complementary key role in the pathogenesis of NASH is played by impaired bile acid signaling, mediated by the FXR and fibroblast growth factor receptor 4 (FGFR4). In patients with NASH, elevated serum levels of bile acids, particularly the FXR antagonist deoxycholic acid, and reduced levels of the agonist chenodeoxycholic acid lead to suppressed FXR signaling [65]. This dysregulation leads to disrupted bile acid metabolism, characterized by increased bile acid synthesis and accumulation, an unfavorable lipid profile with elevated plasma LDL-C and triglycerides, reduced HDL-C levels, and decreased activity of brown adipose tissue [66]. Furthermore, glucose metabolism is also affected, with increased glycolysis and gluconeogenesis, and reduced glycogen synthesis [66]. Reduced serum fibroblast growth factor 19 further impairs FGFR4 signaling, exacerbating hepatic steatosis and inflammation [67]. These findings highlight the interconnected metabolic disruptions and underscore the potential of targeting bile acid metabolism and gut microbiome-driven bile acid conversion as therapeutic strategies for NASH.

We feel that the gut microbiota plays a dual role in liver health, acting both as a promoter of fat accumulation and a driver of inflammatory damage [62,68]. On one hand, certain microbial metabolites, such as SCFAs, can enhance energy harvesting and lipid storage in the liver, contributing to hepatic steatosis [69]. Nevertheless, dysbiosis or an imbalance in the microbial population can lead to the production of pro-inflammatory molecules, such as lipopolysaccharides (LPS) or microbial ethanol, which translocate to the liver through the portal vein, triggering immune activation and chronic inflammation [70]. This duality highlights the complexity of the gut-liver axis, where the microbiota can both sustain metabolic balance and exacerbate liver injury depending on its composition and activity [71]. Furthermore, the diversity and composition of the gut microbiota are not static. Large-scale metagenomic studies reveal extensive unexplored microbial diversity, particularly in non-Westernized populations, where novel species-level genome bins and microbial functions have been uncovered [72]. These findings suggest that changes in lifestyle and geography significantly shape the microbiota, influencing its interaction with host metabolism and inflammatory pathways. This evolving understanding underscores the importance of targeted microbiota interventions to modulate its dual impact—enhancing its metabolic benefits while mitigating its pro-inflammatory potential on liver health.

## 6. Dietary Interventions and Microbiota

**Figure 2:**
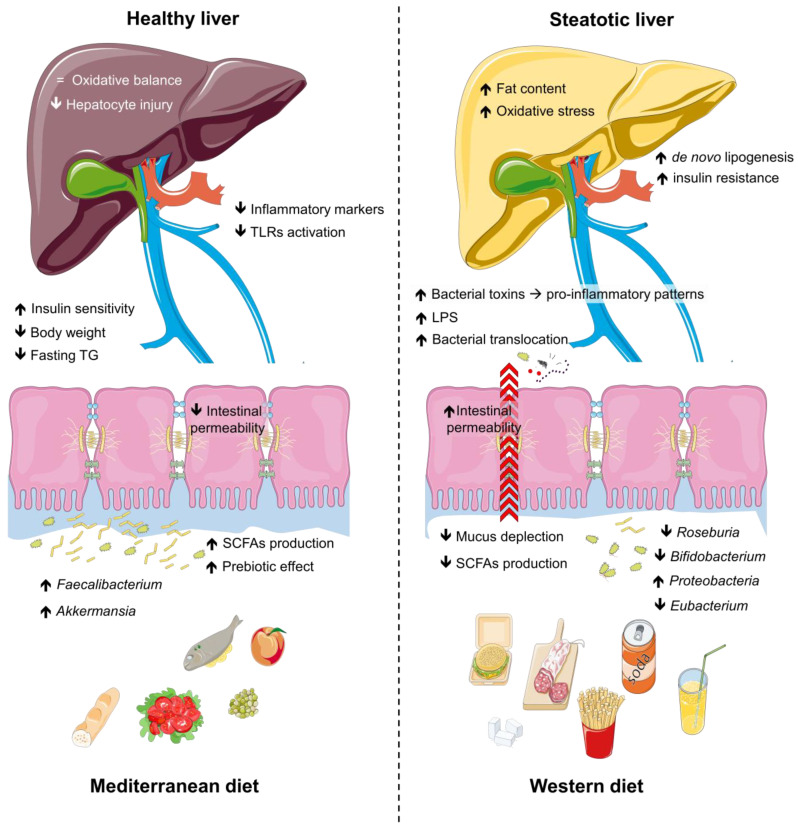
Impact of dietary patterns on gut microbiota, intestinal health, and metabolic outcomes. The Western diet is associated with reduced populations of beneficial gut microbes (e.g., *Bifidobacterium*, *Roseburia*, *Eubacterium*), diminished SCFA production, increased intestinal permeability, and elevated inflammatory markers, promoting lipogenesis, oxidative stress, and bacterial toxin translocation. These changes exacerbate pro-inflammatory pathways, insulin resistance, and hepatocyte injury, leading to metabolic dysfunction and liver steatosis. In contrast, the Mediterranean diet enhances beneficial microbes, SCFA production, and intestinal integrity, while reducing inflammatory markers, body weight, fasting triglycerides, and TLR activation. This fosters oxidative balance, improved insulin sensitivity, and a transition from steatotic to healthy liver states. Abbreviations: LPS, lipopolysaccharides; SCFAs, short-chain fatty acids; TG, triglycerides; TLRs, tool-like receptors.

Dietary habits are increasingly recognized as powerful modulators of gut microbiota composition and functionality [63,73], offering promising strategies for addressing steatotic liver disease [74]. The gut microbiota, shaped by diet quality and composition, plays a key role in metabolic regulation, intestinal health, and immune function [75]. Unhealthy diets rich in saturated fats and refined sugars promote dysbiosis, disrupting intestinal barrier integrity and exacerbating systemic inflammation [76]. In contrast, healthier dietary patterns support microbial diversity and the production of beneficial metabolites such as SCFAs, which exert anti-inflammatory and hepatoprotective effects [77]. To fully understand the nuances of the impact of diet and physical activity on the gut microbiota in steatosis, it is important to explore different dietary patterns, as well as the role of physical activity and the underlying mechanisms in gut microbiota modulation. Figure 2 shows the interplay between dietary patterns and microbiota.

### 6.1. Western Diet

The Western diet, characterized by a high intake of saturated fats, refined sugars, and processed foods, plays a key role in the onset and progression of steatosis and progression to steatohepatitis [78,79,80]. This dietary pattern promotes hepatic fat accumulation by increasing de novo lipogenesis, impairing lipid oxidation, and exacerbating insulin resistance, which are key drivers of liver injury [81]. Recent in vivo research on murine models has demonstrated that the Western diet contributes to the progression of NASH by promoting the gut microbiota-driven production of 2-oleoylglycerol, which activates hepatic stellate cells via several pro-inflammatory pathways, linking *Blautia producta* to liver inflammation and fibrosis [80]. This process is further exacerbated by the Western diet’s ability to increase intestinal permeability [82]. As highlighted by Rohr et al., by disrupting tight junction proteins within the intestinal barrier, this diet allows microbial-derived molecules, such as LPS, to translocate into the portal circulation [83]. These changes could exacerbate systemic inflammation and contribute to the progression of NAFLD. Additionally, the limited dietary fiber typical of the Western diet reduces the production of SCFAs [84], further contributing to the development of NAFLD [85].

Another mechanism involved in the progression of NASH is that the Western diet increases colonic bile acid concentrations, particularly chenodeoxycholic acid, which compromises the integrity of the intestinal epithelial barrier [86]. Additionally, the Western diet fosters the production of pro-inflammatory metabolites by the altered gut microbiota, while additives and emulsifiers present in ultra-processed foods exacerbate inflammation and increase intestinal permeability [87].

Moreover, the overall gut microbiota composition shifts in response to the Western diet, with a decline in beneficial bacterial populations, such as *Bifidobacterium*, *Roseburia*, *Eubacterium*, and *Ruminococcus*, and an overgrowth of pro-inflammatory taxa, including *Proteobacteria* [78]. This reduction in microbial diversity, combined with the dominance of pro-inflammatory microbes, creates a dysbiotic environment that reinforces the negative impact of the diet on liver health and facilitates the phenomenon of “bacterial translocation” [88]. In contrast with other literature evidence, the observed increase in the *Firmicutes/Bacteroidetes* ratio in patients with NAFLD—resembling patterns typically associated with obesity—reveals a contrasting finding that underscores a gap in our understanding of gut dysbiosis and its precise role in disease progression [89].

Through these mechanisms, the Western diet spreads a feedback loop between the gut microbiota and the liver, driving inflammation, metabolic dysregulation, and hepatic injury in NAFLD patients.

### 6.2. Mediterranean Diet

The Mediterranean diet (MD) is widely recognized as the most effective dietary strategy for the prevention and management of liver steatosis, supported by a growing body of evidence highlighting its benefits for liver health [90,91,92,93]. Beyond its hepatoprotective effects, the MD is well-documented in the literature for its ability to significantly improve metabolic parameters [92,94,95,96], regulate blood pressure [97], and reduce inflammatory markers [98], further solidifying its role in the comprehensive management of steatotic liver disease.

The beneficial effects of the MD on steatosis could also be mediated, at least in part, by its impact on gut microbiota [99]. The high intake of dietary fiber from vegetables, legumes, and whole grains promotes the growth of beneficial gut bacteria, which produce SCFAs that enhance the integrity of the intestinal barrier, reduce gut permeability, and limit bacterial translocation into the portal circulation [100]. Furthermore, the polyphenol-rich components of the MD, particularly extra virgin olive oil, also play a crucial role in modulating the gut microbiota, as they act as prebiotics, selectively promoting the growth of beneficial bacteria such as *Faecalibacterium prausnitzii* and *Akkermansia muciniphila*, which are associated with anti-inflammatory effects and improved metabolic health [101,102,103]. A recent observational study concludes that combining physical activity with the MD has a synergistic effect in NAFLD patients, improving metabolic parameters, reducing inflammation, and positively modulating gut microbiota composition [104]. A sub-analysis of the PREvención con DIeta MEDiterránea study has highlighted a relationship between changes in liver disease biochemical indexes and alterations in gut microbiota within the context of a Mediterranean lifestyle. Participants adhering to the MD demonstrated significant reductions in hepatic fat content, fibrosis scores, and liver stiffness. These benefits were strongly associated with positive alterations in gut microbiota composition and reductions in systemic inflammation, emphasizing the critical role of the gut-liver axis in NAFLD management [105].

In summary, the MD was consistently linked to increases in beneficial bacterial genera such as *Faecalibacterium*, which play a key role in maintaining a healthy gut environment and mitigating inflammatory processes linked to the liver [106].

### 6.3. Other Dietary Patterns and Their Potential Impacts on Gut Microbiota and Liver Steatosis

Several dietary patterns have gained attention for their potential role in managing steatotic liver disease by targeting insulin resistance, inflammation, and gut microbiota modulation.

Low-carbohydrate diets (LCDs) have shown effectiveness in improving liver health in NAFLD patients by addressing hepatic fat accumulation, insulin resistance, and gut health [90]. Excessive sugar intake, particularly fructose, drives dietary carbons directly to the liver, promoting de novo lipogenesis [107]. By restricting carbohydrates, LCDs reduce this effect while improving insulin sensitivity by lowering insulin demand [108]. Interestingly, Rad et al. observed that moderate carbohydrate reduction appears to preserve SCFA production, promoting an increase in beneficial taxa such as *Actinobacteria* and a reduction in pro-inflammatory *Proteobacteria* compared to a habitual diet [109].

In this context, very low-calorie diets (VLCDs), which restrict carbohydrate intake to less than 50 g per day and often induce a ketogenic state, have increasingly gained recognition for their potential in managing NAFLD [110]. A recent study by Luukkonen et al. evaluated intrahepatic triglycerides using magnetic resonance spectroscopy in 10 overweight NAFLD patients following a VLCD, resulting in a significant 31% decrease in intrahepatic TG levels and a 57% decrease in the homeostasis model assessment of insulin resistance [111]. However, the restriction of dietary fiber in VLCDs may limit SCFA production [112]. Additionally, the impact of VLCDs on gut microbiota composition remains controversial. Some studies report an increase in beneficial taxa, while pro-inflammatory taxa such as *Enterobacteriaceae* remain stable [113,114]. Conversely, Rondanelli et al. observed a reduction of butyrate-producing bacteria such as *Roseburia* and *Eubacterium rectale* [115]. These shifts may weaken gut integrity, exacerbate endotoxemia, and diminish anti-inflammatory mechanisms, potentially heightening the risk of NAFLD progression.

Moreover, vegetarian diets, which are rich in dietary fiber, polyphenols, and bioactive compounds, address critical pathways in NAFLD, including insulin resistance, oxidative stress, and inflammation [116]. Polyphenols—abundant in plant-based foods—improve liver health and reduce progression to NASH by selectively enhancing beneficial bacteria such as *Bifidobacterium* and *Lactobacillus* in vivo and in vitro studies [117]. Furthermore, vegetarian diets promote a varied gut microbiota, increasing SCFA production [118,119]. Additionally, their low levels of saturated fats further enhance their hepatoprotective effects [120].

Similarly, the Nordic diet emphasizes whole grains such as rye and oats, along with fatty fish rich in omega-3 fatty acids [121,122]. Notably, Landberg et al. have highlighted that the Nordic diet positively influences gut microbiota composition with significant metabolic benefits, promoting beneficial species such as *Bifidobacterium*, *Lactobacillus*, *Roseburia*, and *Faecalibacterium prausnitzii*, while reducing pro-inflammatory taxa such as *Proteobacteria* and *Enterobacteriaceae* [121].

Another dietary pattern showing promising potential for managing NAFLD is intermittent fasting (IF). This approach alternates periods of eating and fasting, during which insulin levels decrease, promoting fatty acid oxidation and suppressing de novo lipogenesis [123]. Moreover, IF enhances gut microbiota diversity, encouraging the growth of beneficial taxa such as *Lachnospiraceae*, which produce butyric acid to reinforce gut barrier integrity and mitigate systemic inflammation [124]. However, a recent systematic review highlighted the variability in microbiota changes induced by IF, showing limited or inconsistent improvements in microbial diversity and composition [125]. Additionally, prolonged fasting may disrupt circadian rhythms and exacerbate gut dysbiosis in sensitive individuals, potentially undermining its benefits for NAFLD management [126].

Despite their distinct characteristics, these dietary patterns share a common ability to enhance metabolic health, improve gut microbiota composition, and strengthen the gut-liver axis. To fully harness their benefits and address specific limitations, individualized dietary approaches remain essential.

## 7. Microbiota-Based Therapeutics

Emerging microbiota-based therapies offer innovative strategies to restore gut microbial balance and address a wide range of health conditions. These approaches include probiotics, which introduce beneficial live microorganisms to promote a healthier microbial ecosystem, and prebiotics, which serve as substrates to selectively stimulate the growth of advantageous bacteria [127,128,129]. Fecal microbiota transplantation (FMT) has demonstrated significant efficacy in restoring microbial diversity, particularly in cases such as *Clostridium difficile* infections, and is being explored for metabolic and liver disorders [130,131]. Additionally, the precision modulation of microbial metabolites, such as SCFAs and bile acids, targets specific pathways to directly influence disease mechanisms [129]. Together, these therapies highlight the transformative potential of microbiota-based interventions in managing complex, multifactorial diseases.

In this field, FMT has been initially recognized for its efficacy in treating recurrent Clostridioides difficile infections, with success rates exceeding 80% [132]. Over time, its application field has broadened to include a range of complex diseases, such as colorectal cancer, inflammatory bowel diseases, and metabolic disorders [133]. To note, FMT has also emerged as a promising therapeutic strategy for managing steatotic liver disease, particularly through its impact on gut microbiota composition and function. Clinical studies demonstrated that FMT from healthy donors can modulate gut microbiota, improve gut barrier integrity, and alter plasma metabolites, thereby influencing hepatic inflammation and lipid metabolism [134,135]. Recent trials have highlighted the efficacy of FMT in reducing liver fat and improving the histological markers of necroinflammation in NAFLD patients [131,134,135]. An allogenic FMT using stool from lean, vegan donors resulted in significant changes in hepatic gene expression and plasma metabolites, correlating with reduced liver inflammation [134]. Similarly, another randomized trial showed that FMT decreased hepatic fat attenuation and restored microbial diversity, particularly in lean NAFLD patients, indicating that donor and recipient characteristics influence therapeutic outcomes [131]. Although FMT is generally well-tolerated and has demonstrated potential, further research is required to standardize protocols and elucidate the mechanisms underlying its effects in NAFLD.

Recently, another context was studied in which bacteriophage therapy was explored as a novel intervention for fatty liver disease caused by high alcohol-producing *Klebsiella pneumoniae* (HiAlc Kpn) [136]. This specific strain exacerbates liver steatosis by producing endogenous alcohol, which disrupts hepatic lipid metabolism and induces inflammation. The study demonstrated that a targeted bacteriophage effectively eradicated HiAlc Kpn in murine models without significantly altering the broader gut microbiota. Phage treatment not only alleviated hepatic steatosis and inflammation but also restored lipid and carbohydrate metabolism. Importantly, this approach showed no significant side effects or dysbiosis, positioning bacteriophage therapy as a promising alternative to traditional antibiotic treatments for liver diseases linked to microbial dysbiosis [136].

We believe that precision microbiota-based therapies offer targeted solutions for complex diseases such as liver steatosis. While effective in modulating gut microbiota and reducing inflammation, challenges remain. FMT and bacteriophage therapy face hurdles in donor variability, standardization, and potential resistance development. The specificity of these approaches minimizes side effects but requires precise pathogen identification. Despite these limitations, their potential for personalized, minimally invasive treatments makes them a promising frontier in managing liver and metabolic diseases.

## 8. Challenges and Knowledge Gaps

The study of gut microbiota and its relationship with liver diseases faces several significant challenges and knowledge gaps that hinder the consistency and applicability of findings. These issues range from demographic variability to technological limitations, complicating the ability to draw definitive conclusions and translate insights into clinical practice [137].

Demographic diversity introduces inherent variability, such as differences in sex, age, and ethnicity. While such diversity is essential for generalizability, it introduces variability that can hide specific microbiota-related patterns. Hormonal influences, age-related microbial shifts, and genetic predispositions further complicate analyses, making it difficult to identify universal signatures associated with liver diseases. Moreover, geographical location plays a pivotal role in shaping the gut microbiota. Variations in ethnicity, cultural practices, and dietary habits across regions such as North America, Asia, and Europe result in distinct microbiota compositions, complicating cross-study comparisons and raising questions about the global applicability of findings derived from specific populations [138].

Another major challenge lies in the strong association between the gut microbiota and metabolic disorders such as obesity and type 2 diabetes mellitus, which frequently coexist with NAFLD. This overlap makes it difficult to isolate microbiota signatures specific to NAFLD, as many of the microbial changes observed may reflect these comorbid conditions rather than liver-specific pathophysiology. Further complicating the issue, the gut microbiota is highly sensitive to medications, such as antibiotics, proton pump inhibitors, and antidiabetic drugs, which can substantially alter its composition. Many studies do not adequately account for medication use, introducing variability that compromises the interpretation of results. Ensuring that observed microbiota changes are genuinely related to liver health rather than pharmacological interventions remains a critical challenge [139].

In addition to these biological and clinical complexities, differences in gut microbiota sequencing technologies and bioinformatics pipelines further contribute to variability in results. Variations in DNA extraction methods, sequencing platforms, and data analysis approaches lead to discrepancies in microbial composition and functional profiling [140]. This technological variability limits reproducibility and comparability across studies, emphasizing the urgent need for standardized methodologies in gut microbiota research [141].

Addressing these challenges requires concerted efforts to harmonize study designs, including consistent demographic and clinical data collection, and to develop multicenter studies that account for geographical and population-based variability. Refining analytical approaches with advanced bioinformatics tools can help disentangle the effects of comorbidities and medication use [142,143]. Furthermore, the adoption of standardized sequencing technologies and data analysis pipelines is essential to improve reproducibility and foster a deeper and more reliable understanding of the gut-liver axis in NAFLD [142,144,145].

## 9. Conclusions

This review highlights the intricate interplay between the gut microbiota and steatotic liver disease, emphasizing the gut-liver axis as a critical mediator of health and disease. The evidence presented underscores the dual role of microbiota in either promoting metabolic balance or exacerbating liver inflammation, depending on its composition and activity. Emerging dietary and microbiota-based interventions, such as the MD and FMT, offer promising avenues for the targeted management of liver diseases. However, significant challenges remain, including variability in patient populations, study designs, and technological methodologies. Future research should focus on integrating multi-omics approaches and harmonizing study protocols to unravel the complexities of the gut-liver axis. By advancing our understanding of these relationships, we can cover the way for personalized and effective strategies to combat the rising global burden of steatotic liver disease.

## Data Availability

No new data were created or analyzed in this study.
